# Transcriptomic and metabolomic analyses provide insights into the biosynthesis of chlorogenic acids in *Lonicera macranthoides* Hand.-Mazz

**DOI:** 10.1371/journal.pone.0251390

**Published:** 2021-05-26

**Authors:** Yuan Pan, Xiao Zhao, Xiao-li Wu, Yu Wang, Jun Tan, Da-xia Chen

**Affiliations:** 1 Chongqing Academy of Chinese Materia Medica, Chongqing, China; 2 Chongqing Engineering Research Center for Fine Variety Breeding Techniques of Chinese Materia Medica, Chongqing, China; 3 Chongqing Sub-Center of National Resource Center for Chinese Materia Medica, China Academy of Chinese Medical Science, Chongqing, China; Universite d’Orleans, FRANCE

## Abstract

*Lonicera macranthoides* Hand.-Mazz (*L*. *macranthoides*) is a medicinal herb that is widely distributed in South China. The developmental stage and corolla dehiscence of the flower are the important factors affecting the quality of medicinal ingredients. However, neither the regulatory mechanism controlling chlorogenic acids biosynthesis in *L*. *macranthoides* nor the molecular basis of effect of corolla dehiscence on the quality of medicinal materials is fully understood. In this study, metabolomics and transcriptomics were used to analyze the metabolic and transcriptional differences of two different cultivars closed bud type (Bt), and flowering type (Ft), as well as the effect of jasmonic acid methyl ester (MeJA) on chlorogenic acids (CGAs) biosynthesis. In total, large number of differentially expressed genes (DEGs) and differentially accumulated metabolites (DAMs) were filtered among three lines of samples. Gene metabolite correlation analyses revealed a ‘core set’ of 30 genes and 54 genes that were strongly correlated with CGAs biosynthesis and regulating the flowering, respectively. Quantitative real-time polymerase chain reaction results proved the alterations in the expression levels of genes encoding the pathways involved in CGAs biosynthesis. The ion abundances of CGAs were most significantly increased, while some of the CGAs derived and Caffeoyl-CoA-derived substances showed the most largely reduced abundances in the closed bud type (Bt) compared to the flowering type (Ft). MeJA may leads to the activation of downstream genes in CGAs biosynthesis pathway. Overall, there were significant differences in the transcriptional and metabolic levels of CGAs biosynthesis pathway in flower buds of different flowering cultivars. The redirection of metabolic flux may contribute to increased accumulation of CGAs. However, whether MeJA and flowering have direct effects on the accumulation of CGAs needs further studied. These researches effectively expanded the functional genomic library and provide new insights into CGAs biosynthesis in *L*. *macranthoides*.

## Introduction

*Lonicera*. *macranthoides* Hand. Mazz (*L*. *macranthoides*), belonging to the Caprifoliaceae family, is a stolon shrub featuring perennial, evergreen and twining vines, which are distributed widely in South China and are often used in traditional Chinese medicine. *L*. *macranthoides* is registered as “mountain honeysuckle” in the 2010 *Chinese Pharmacopoeia* [1]. In light of the preventive and therapeutic roles of *L*. *macranthoides* in severe acute respiratory syndrome (SARS, 2003), H1N1 (2009) and COVID-19 (2019) outbreaks in China, the demand for *L*. *macranthoides* has increased gradually in recent years [2, 3]. The dry floral buds or open flowers are the most important medicinal tissues that have been recorded in the classical pharmacopeia of Chinese traditional medicine “Ming Yi Bie Lu” and “Shen Nong Ben Cao Jing” [4], which are used for the prevention and treatment of severe acute respiratory syndromes, H1N1 influenza, and hand-foot-and-mouth disease [5, 6]. A variety of phenolic acids, flavonoids, volatile oils and saponins were detected in the floral buds or flowers of *L*. *macranthoides*. They display multiple biological activities, including antibacterial, anti-inflammatory, antioxidant, antiviral, antiangiogenic, antipyretic, hepatoprotective and antitumor effects [6–11].

Among all bioactive components, chlorogenic acids (CGAs) are the important compound in plants, which is composed of some trans-cinnamic acids in the ester family, such as caffeic acid, ferulic acid and quinic acid. More than 30 different species of chlorogenic acids have been identified in different plants [12]. CGAs accumulate in the flowers, stems and leaves of *L*. *macranthoides*, especially in flowers [13, 14]. They are considered to have many important biological activities, such as high antioxidant activity, for the removal of harmful free radicals and prevention activities [12, 15–18].

CGAs are the kind of phenylpropanoid compound produced by shikimic acid pathway during aerobic respiration. The molecular mechanism underlying CGAs accumulation has been extensively studied in numerous plants. In this pathway, many active biological enzymes are involved, such as phenylalanine ammonia lyase (PAL) [19], 4-coumarate coenzyme A ligase (4CL) [20], cinnamate 4-Hydroxylase (C4H), hydroxycinnamoyl transferase (HCT) [21] and coumarate 3-hydroxylase (C3H) [22, 23]. When the phenylalanine ammonia-lyase gene (*PAL*) was transferred into tobacco, the transcription level of *PAL* was improved, and the content of chlorogenic acids in the leaves of transgenic plants was also significantly increased, which showed that *PAL* was a key gene in the CGAs biosynthesis pathway [19]. The coumarate 3-hydroxylase (C3H) protein of *Arabidopsis thaliana* is involved in the metabolism of CGAs. It can hydroxylate the third position of coumaric acid and play a role in the esterification of coumaric acid into shikimic or quinic acid [22, 23]. HCT is a key enzyme acting downstream of the CGAs metabolic pathway and plays a role in the hydroxylation of coumaroyl CoA to caffeyl-CoA [24, 25]. In the preliminary study, three different CGAs synthesis pathways were hypothesized. First, CGAs is generated from caffeyl CoA and quinic acid via catalysis by hydroxycinnamoyl-CoA quinate hydrocycinnamoyl transferase (*HQT)* [20]. Second, the synthesis was catalyzed by *HCT* and then hydrolyzed by *C3H* to obtain coumaroyl quinine [26]. The third pathway was the caffeic acid glucosidase, which serves as the active intermediate [27]. There may be different biosynthetic pathways in different species, or the importance of different biosynthetic pathways varies in different species.

Recent technical advancements in transcriptome and metabolome analyses have provided effective ways to identify new genes and metabolites and to elucidate complex secondary metabolic bioprocesses in plants [28]. Comparative metabolomic analysis reveals distinct flavonoid biosynthesis regulation for leaf color development of Cymbidium sinense ‘Red Sun’ [29]. MeJA might upregulate the expression of upstream genes in the flavonoid biosynthesis pathway and downregulate the expression of downstream genes, thus promoting the biosynthesis of quinone chalcones in sunflower [30]. However, whether MeJA has any effect on the synthesis of CGAs in plants has not been reported.

**Fig 1:**
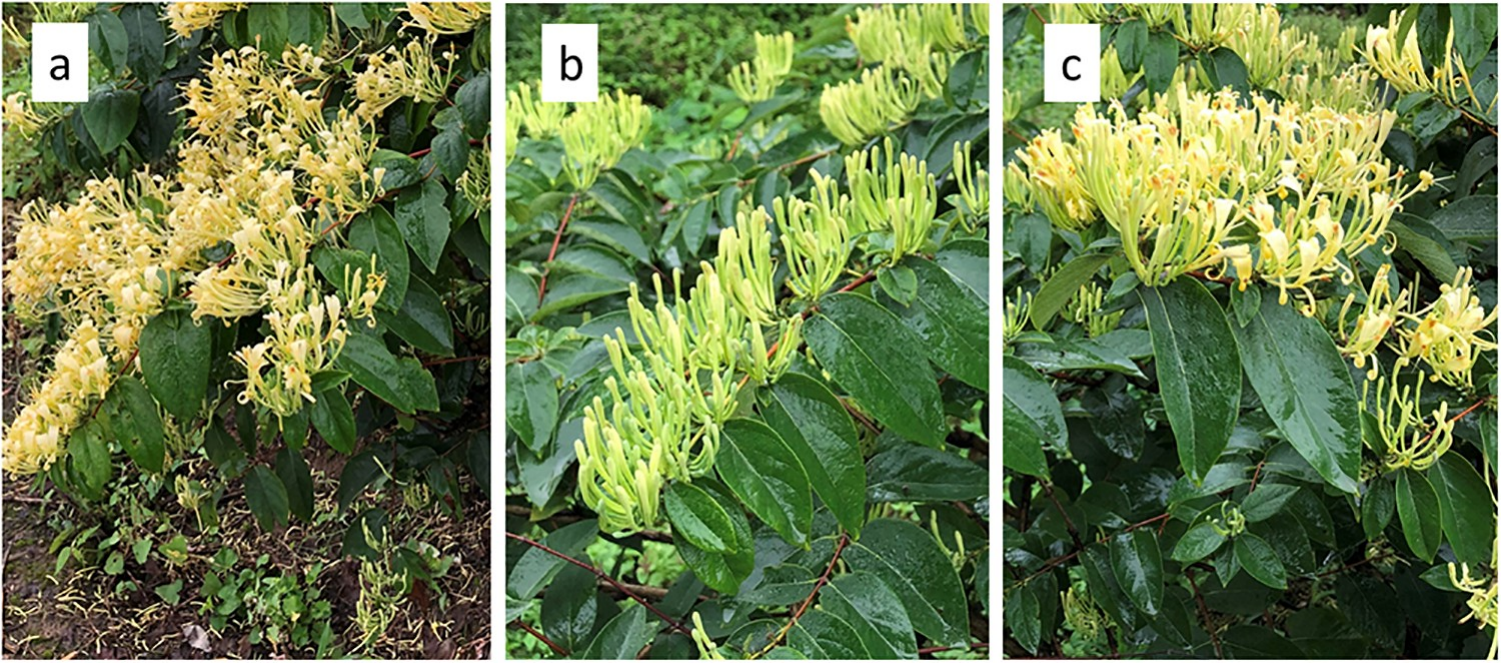
Image shows the different types of *Lonicera*. *macranthoides* Hand.-Mazz (*L*. *macranthoides*). (a): The flowering type (Ft). (b): The closed bud type (Bt). (c): The MeJA-treated closed bud type (Bt1000).

*L*. *macranthoides* is widely distributed in South China and is highly adaptable to growth, but evolution has led to significant changes in yield and quality. For a long time, the short bud period and inability to pick buds in time were problems in the production and application of *L*. *macranthoides*. In this study, flowering type (Ft), closed bud type (Bt) and MeJA-treated closed bud type (Bt1000) (Fig 1) were used as experimental materials to conduct transcriptomic and metabolome comparative analysis of flowers to identify candidate genes involved in CGAs biosynthesis. Because of MeJA can effectively regulate corolla opening, we used MeJA-treated closed bud type (Bt1000) to test whether flowering was related to the biosynthesis of CGAs. This is the new attempt to investigate the transcriptional and metabolite differences between the two different flowering cultivars of *L*. *macranthoides*, as well as the effect of flowering on chlorogenic acids biosynthesis, and the results will enhance our understanding of the metabolic mechanisms and molecular basis of CGAs biosynthesis in *L*. *macranthoides*.

## Materials and methods

### Plant materials

Ft’ is a flowering type of *L*. *macranthoides* that is a local conventional cultivar in Chongqing, China. ‘Bt’ is a closed bud type of *L*. *macranthoides* that was bred from ‘Ft’ and widely cultivated in Chongqing, China. The corolla of Bt does not crack until it falls, and the Ft’s bud period was shorter, followed by corolla cracking. ‘Ft’ and ‘Bt’ were planted in parallel and were all 8 years old. The experiment was conducted in the southern hilly region of Banan District, Chongqing Municipality, China (N29.34, E106.92). On sunny days, we collected yellow white buds (Ft, preflowering period) from Ft. In Bt, the yellow white buds were collected first, and then the remaining yellow white buds in the same branch were sprayed with MeJA (1000 ppm) by miniature sprayer. After 24 hours, the opened flowers (MeJA-treated closed bud type, Bt1000) were collected and used as samples. Three biological replicates were performed in each case. After washing, the samples were immediately frozen in liquid nitrogen and stored at -80°C until further use in metabolite, RNA sequencing (RNA-Seq), and qPCR analyses.

### Sample preparation and extraction

The sample preparation, extract analysis, metabolite identification and quantification were performed at Wuhan MetWare Biotechnology Co., Ltd. (www.metware.cn) following their standard procedures and previously fully described by Yuan et al. (2018) and Wang et al. (2017) [31, 32]. The freeze-dried sample was crushed using a mixer mill (MM 400, Retsch, Germany) with a zirconia bead for 1.5 min at 30 Hz. One hundred milligrams of powder was weighed and extracted overnight at 4°C with 1.0 mL 70% aqueous methanol. Following centrifugation at 10 000 × g for 10 min, the extracts were absorbed (CNWBOND Carbon-GCB SPE Cartridge, 250 mg, 3 mL; ANPEL, Shanghai, China, www.anpel.com.cn/cnw) and filtered (SCAA-104, 0.22 μm pore size; ANPEL, Shanghai, China, http://www.anpel.com.cn/) before LC-MS analysis.

### UHPLC and ESI-Q TRAP-MS/MS conditions

LIT and triple quadrupole (QQQ) scans were acquired on a triple quadrupole-linear ion trap mass spectrometer (Q TRAP), API 4500 Q TRAP LC/MS/MS System, equipped with an ESI Turbo Ion-Spray interface, operating in positive ion mode and controlled by Analyst 1.6.3 software (AB Sciex). The ESI source operation parameters were as follows: ion source, turbo spray; source temperature 550°C; ion spray voltage (IS) 5500 V; ion source gas I (GSI), gas II (GSII), and curtain gas (CUR) were set at 55, 60, and 25.0 psi, respectively; and the collision gas (CAD) was high. Instrument tuning and mass calibration were performed with 10 and 100 μmol/L polypropylene glycol solutions in QQQ and LIT modes, respectively. QQQ scans were acquired as MRM experiments with collision gas (nitrogen) set to 5 psi. DP and CE for individual MRM transitions were performed with further DP and CE optimization. A specific set of MRM transitions was monitored for each period according to the metabolites eluted within this period.

### Metabolite identification and quantification

Metabolite data analysis was conducted with Analyst 1.6.1 software (AB SCIEX, Ontario, Canada). The supervised multivariate method, partial least squares-discriminant analysis (PLS-DA), was used to maximize the metabolome differences between a pair of samples. The relative importance of each metabolite to the PLS-DA model was checked using the parameter called variable importance in projection (VIP). Metabolites with VIP ≥ 1 and fold change ≥2 or fold change ≤0.5 were considered differential metabolites for group discrimination [31].

### RNA sequencing and annotation

Total RNA isolation and purification and cDNA library construction and sequencing were performed as previously described [32, 33]. Total RNA was prepared by using the TRIzol reagent (Invitrogen) following the manufacturer’s protocol. Total RNA purity and concentration were determined using the NanoPhotometer^®^ spectrophotometer (IMPLEN, CA, USA) and the Qubit^®^ RNA Assay Kit in Qubit^®^ 2.0 Fluorometer (Life Technologies, CA, USA). mRNA was isolated from total RNA using magnetic beads with oligo (dT) primers; cDNA was synthesized using a cDNA synthesis kit (TaKaRa, Dalian, China) and linking the sequencing adapter to both ends. The library preparations were sequenced on an Illumina HiSeq 4000 platform. Transcriptome assembly was accomplished based on the left.fq and right.fq using Trinity [34, 35] with min_kmer_cov set to 2 by default and all other parameters set default. Gene function was annotated based on the following databases: Nr (NCBI nonredundant protein sequences); Nt (NCBI nonredundant nucleotide sequences); Pfam (Protein family); KOG/COG (Clusters of Orthologous Groups of proteins); Swiss-Prot (A manually annotated and reviewed protein sequence database); KEGG (Kyoto Encyclopedia of Genes and Genomes Ortholog database) [36]; and GO (Gene Ontology) [37]. The fragments per kilobase million (FPKM) were calculated to quantify gene expression [38], and the DESeq2 R package [39] was adopted to determine the differentially expressed genes (DEGs). The differentially expressed genes were identified with the following parameters: corrected P-value of 0.05 and absolute fold change ≥2. GO and KEGG enrichment analyses of differentially expressed genes were further implemented employing the clusterProfiler R package [40].

### Quantitative Real-Time PCR (qRT-PCR) analysis

For the expression analysis of the DEGs, total RNA was extracted with TRIzol reagent (Invitrogen, CA, USA) from 100 mg of all the samples. First-strand cDNA was synthesized from 2 μg of total RNA using M-MLV Reverse Transcriptase (Promega, WI, USA) according to the manufacturer’s instructions. The reactions (20 μL) were terminated after 40 cycles using the CFX96TM Real-Time PCR Detection System (Bio-Rad, CA, USA), and the 18S reference gene was used as an internal control. All of the assays were repeated at least three times. The relevant PCR primer sequences are shown in S1 Table and were designed based on the CDS and ESTScan sequences of *L*. *macranthoides* by Primer Express Software (Applied Biosystems, CA, USA).

### Correlation analysis between metabolome and transcriptome data

Pearson’s correlation coefficients were calculated between the metabolome and transcriptome data. The coefficients were calculated from log2 (fold change) of each metabolite and log2 (fold change) of each transcript with the Excel program. Correlations with a coefficient of R2 > 0.8 were selected.

### Sequence accession numbers

The whole set of transcript data can be found in the National Center for Biotechnology Information (NCBI) SRA database (SRR12727863- SRR12727871).

## Results

### Plant secondary metabolomics analysis

**Fig 2:**
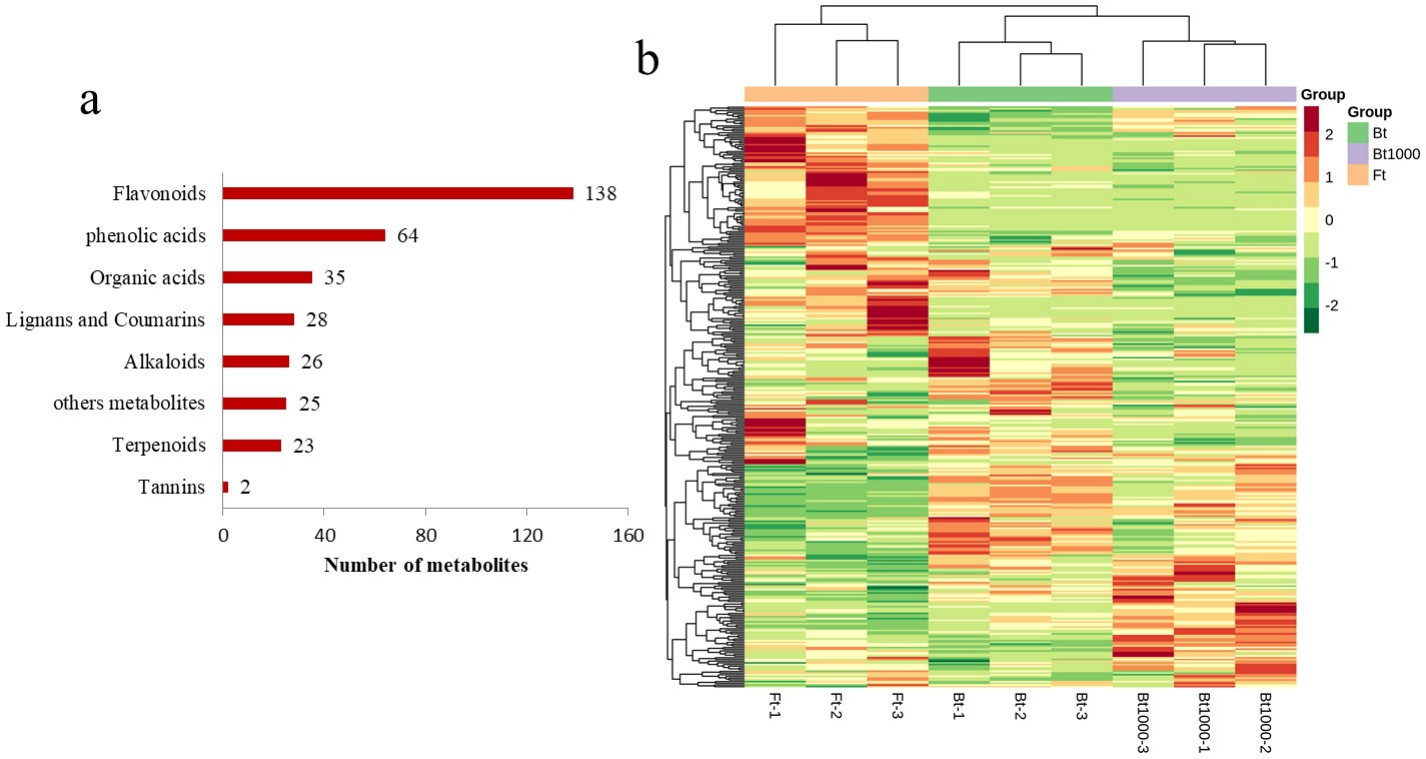
Number and heat map of metabolite profiles of different varieties. (a) Numbers of different types of metabolites in all samples. (b) Clustering heat map of the metabolites detected in the total samples. Each example is visualized in a single column, and each metabolite is represented by a single row. Red indicates high abundance, whereas low relative metabolites are shown in green. Ft indicates the flowering type, Bt indicates closed bud type and Bt1000 indicates MeJA-treated closed bud type (Bt1000).

In general, the best harvesting period for *L*. *macranthoides* was the yellow white bud stage. In this study, the flowering type (Ft), closed bud type (Bt) and MeJA-treated closed bud type (Bt1000) were used to monitor changes in the metabolite abundance associated with the biosynthesis of active substances in *L*. *macranthoides* (Fig 2). We described the metabolome of the three lines of samples using the widely targeted metabolomics approach. In total, 341 metabolites were detected and quantified from *L*. *macranthoides*, including 64 phenolic acids, 138 flavonoids, 28 lignans and coumarins, 2 tannins, 26 alkaloids, 23 terpenoids, 35 organic acids, and 25 other metabolites (Fig 2a). In the clustering heat map, the accumulation of metabolites displayed clear phenotypic variation in terms of the pattern of metabolite abundance in different types, and the Ft was clearly distinct from the Bt (Fig 2b). In this study, the concentration data of metabolites were used for the hierarchical heat map clustering analysis of samples. We observed that all the biological replicates were grouped together (top side of the figure), indicating a high reliability of the metabolome data (Fig 2b). In particular, we observed clear separations among Ft, Bt and Bt1000. The above indicates that there are obvious differences in the metabolic characteristics of them.

### Comparison of metabolites produced by three lines of samples (Bt, Ft, Bt1000)of *L*. *macranthoides*

**Fig 3:**
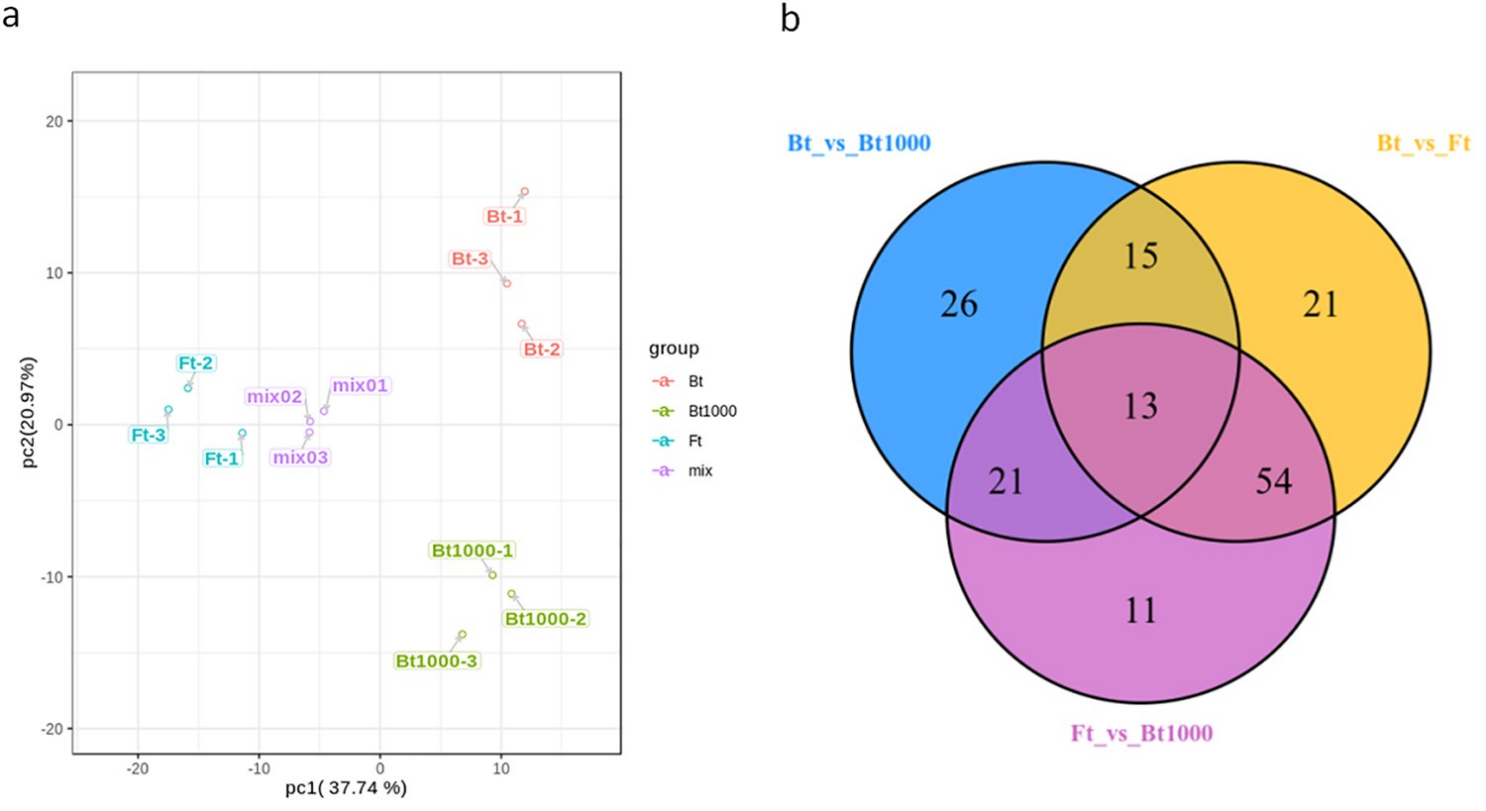
Multivariate analysis of metabolic profiling. (a): principal components analysis (PCA) of metabolites. (b): Venn diagram representing the numbers of differentially accumulated metabolites (DAMs). Ft indicates the flowering type, Bt indicates closed bud type and Bt1000 indicates MeJA-treated closed bud type (Bt1000).

**Fig 4:**
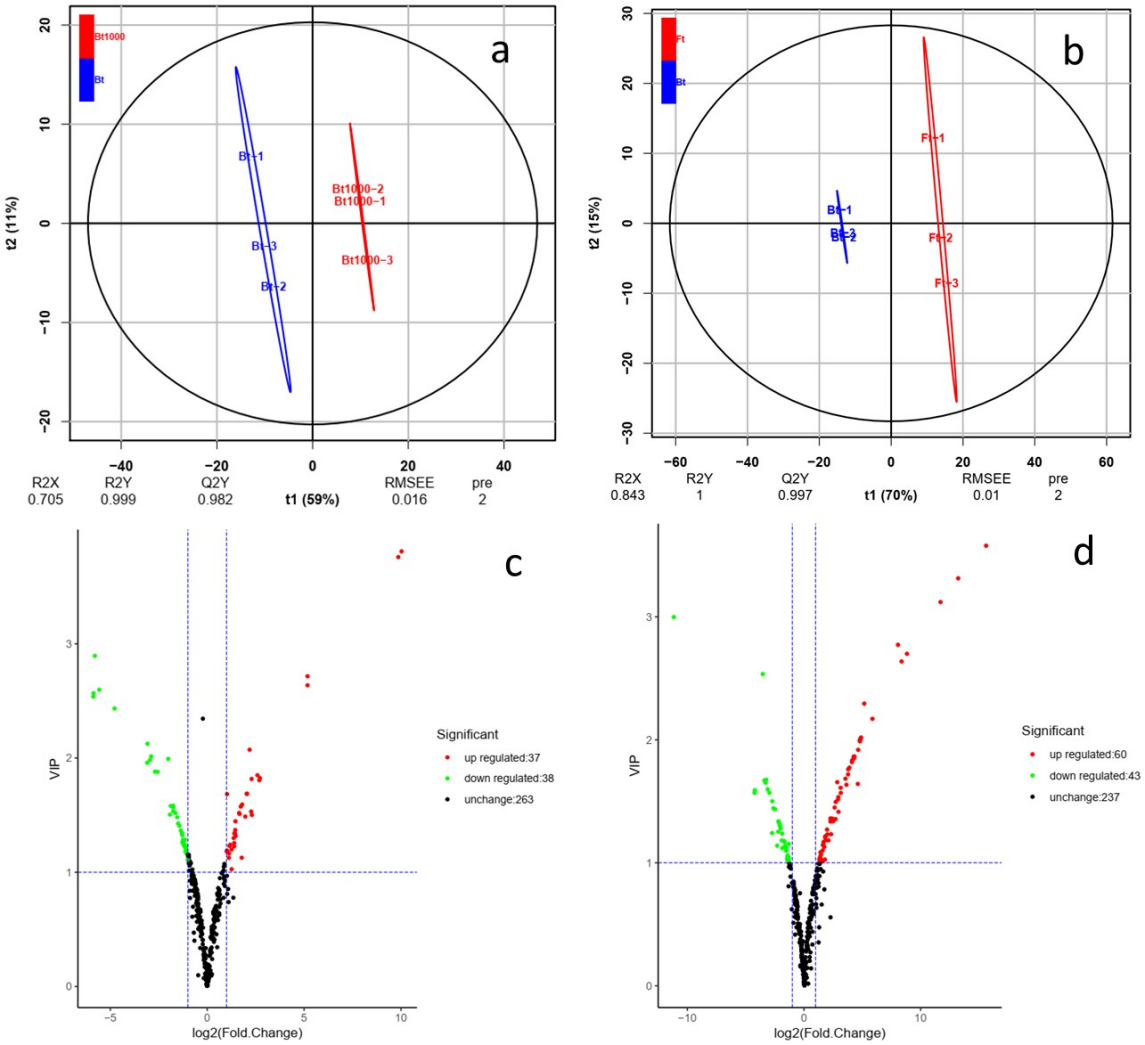
Differential metabolite analysis. (a): Orthogonal partial least squares discriminant analysis (OPLS-DA) score plot. Blue dots indicate Bt, and red dots indicate Bt1000. (b): OPLS-DA score plot. Blue dots indicate Bt, and red dots indicate Ft. (c): Volcano plot of differential metabolites between Bt and Bt1000. (d): Volcano plot of differential metabolites between Bt and Ft. Each point in the volcanic plot represents a metabolite, the abscissa represents the logarithm of the quantitative difference multiples of a metabolite in two samples, and the ordinate represents the variable importance in project (VIP) value. The green dots in the figure represent downregulated differentially expressed metabolites, the red dots represent upregulated differentially expressed metabolites, and the black dots represent detected metabolites but that are not significantly different. Ft indicates the flowering type, Bt indicates closed bud type and Bt1000 indicates MeJA-treated closed bud type (Bt1000).

To explore the changes in secondary metabolites in Bt, Ft, Bt1000, principal component analysis (PCA) and orthogonal partial least-squares discrimination analysis (OPLSDA) were applied to the metabolites, and the three lines of samples showed significant separations (Figs 3 and 4). From the results, they were clearly distinguished in the PC1 dimension of the PCA score graph (37.74% variation) and were further differentiated at the PC2 dimension, indicating that the secondary metabolites varied significantly with different types and treatments. The OPLS-DA model compared the entire metabolite content of the core samples in pairs to evaluate the differences between Bt1000 and Bt (R^2^Y = 1, Q^2^Y = 0.986; Fig 4a) and between Bt and Ft (R^2^Y = 1, Q^2^Y = 0.998; Fig 4b). High predictability (Q2) of the OPLSDA models was stable and reliable and could be used to further screen for differential metabolites. The differentially accumulated metabolites (DAMs) between pairs of samples (Bt vs Bt1000 and Bt vs Ft) were determined based on the variable importance in projection (VIP) ≥ 1 and fold change ≥ 2 or fold change ≤0.5 [41]. There were 75 significantly different metabolites between Bt and Bt1000 (Bt1000 had 38 downregulated and 37 upregulated metabolites) and 103 between Bt and Ft (Ft had 43 downregulated and 60 upregulated metabolites) (Fig 4c and 4d). Furthermore, 13 common differential metabolites were detected in all three comparison groups (Fig 3b). For Ft vs Bt, 103 DAMs were detected and quantified, including 12 phenolic acids, 62 flavonoids, 12 lignans and coumarins, 1 tannins, 2 alkaloids, 2 terpenoids, 7 organic acids, and 5 others. The metabolite with the largest fold change was isorhamnetin 3-O-neohesperidoside and the top 20 fold change metabolites were mainly Flavonoids (S9 Table). For Bt vs Bt1000, 75 DAMs were detected and quantified, including 11 phenolic acids, 26 flavonoids, 5 lignans and coumarins, 2 tannins, 9 alkaloids, 2 terpenoids, 10 organic acids, and 10 others. The metabolite with the largest fold change was Nomilin and the top 20 fold change metabolites mainly fell in other categories, mainly Flavonoids and Organic acids (S10 Table).

**Fig 5:**
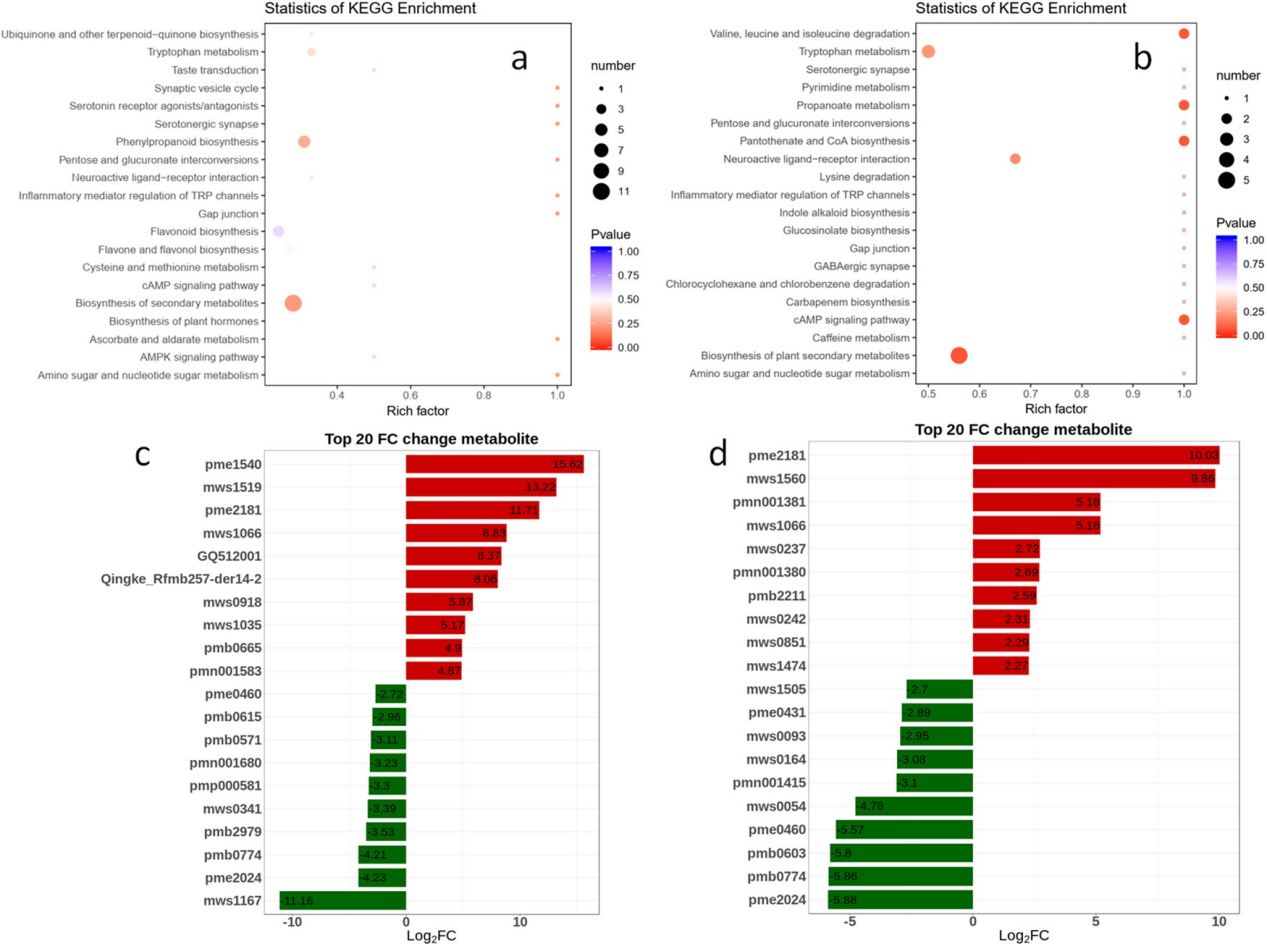
Identification and functional characterization of differentially accumulated metabolites (DAMs). Kyoto Encyclopedia of Genes and Genomes Ortholog database (KEGG) enrichment analysis of the DAMs between (a) Ft and Bt and between (b) Bt1000 and Bt. Top 20 metabolite changes between (c) Ft and Bt and between (d) Bt1000 and Bt. Ft indicates the flowering type, Bt indicates closed bud type and Bt1000 indicates MeJA-treated closed bud type.

KEGG is the main public database of metabolic pathways and is used for the research of genes, expression information, and metabolite content in a general network. The top enriched KEGG terms among the DAMs detected for Bt vs Ft were biosynthesis of secondary metabolites, phenylpropanoid biosynthesis and tryptophan metabolism (Fig 5a). Different from Bt vs Ft, the top enriched KEGG terms among the DAMs detected for Bt vs Bt1000 were biosynthesis of secondary metabolites, tryptophan metabolism and neuroactive ligand-receptor interaction (Fig 5b).

### Transcriptome profiles and major transcriptomic differences

To investigate the transcriptomic changes among Bt, Ft, Bt1000, nine cDNA libraries were generated and sequenced using an Illumina deep-sequencing HiSeq^™^ 4000. Among all the raw reads, 97% had Phred-like quality scores at the Q20 level (an error probability of 1%). After removing low-quality sequences, 424, 085, 868 clean reads and 63.63 GB clean bases were used to assemble the transcriptome data using the Trinity method (Table 1). After assembly, we obtained 101 214 unigenes. The unigenes were annotated based on seven public databases [i.e., NR, NT, KEGG, Swiss-Prot, PFAM, GO and KOG/COG]. In total, 95, 999 unigenes (94.84%) were annotated in at least one database, with 22, 472 (22.2%) unigenes being annotated in all databases (Table 2).

**Table 1:** Summary of the sequence analyses.

Sample	Raw Reads	Clean reads	Clean bases (G)	Error(%)	Q20(%)	Q30(%)	GC(%)
Bt_1	55387322	54472426	8.17	0.01	97.45	93.37	43.84
Bt_2	45383098	44708378	6.71	0.02	97.39	93.22	43.99
Bt_3	44197522	42787210	6.42	0.02	96.96	92.56	44.11
Bt1000_1	43989376	43307912	6.50	0.01	97.53	93.56	44.07
Bt1000_2	51287894	50447500	7.57	0.02	97.4	93.25	43.87
Bt1000_3	43990878	43384612	6.51	0.02	97.37	93.15	43.8
Ft_1	51014560	50039774	7.51	0.02	97.33	93.15	44.47
Ft_2	49611964	48823626	7.32	0.02	97.36	93.14	43.89
Ft_3	46886456	46114430	6.92	0.02	97.38	93.18	43.79

Note: Ft indicates the flowering type, Bt indicates closed bud type and Bt1000 indicates MeJA-treated closed bud type.

**Table 2:** The success rate of unigene annotation.

Database	Number of Unigenes	Percentage (%)
Annotated in NR	91593	90.49
Annotated in NT	78383	77.44
Annotated in KO	43098	42.58
Annotated in SwissProt	80989	80.01
Annotated in PFAM	77350	76.42
Annotated in GO	77885	76.95
Annotated in KOG	33123	32.72
Annotated in all Databases	22472	22.2
Annotated in at least one Database	95999	94.84
Total Unigenes	101214	--

**Fig 6:**
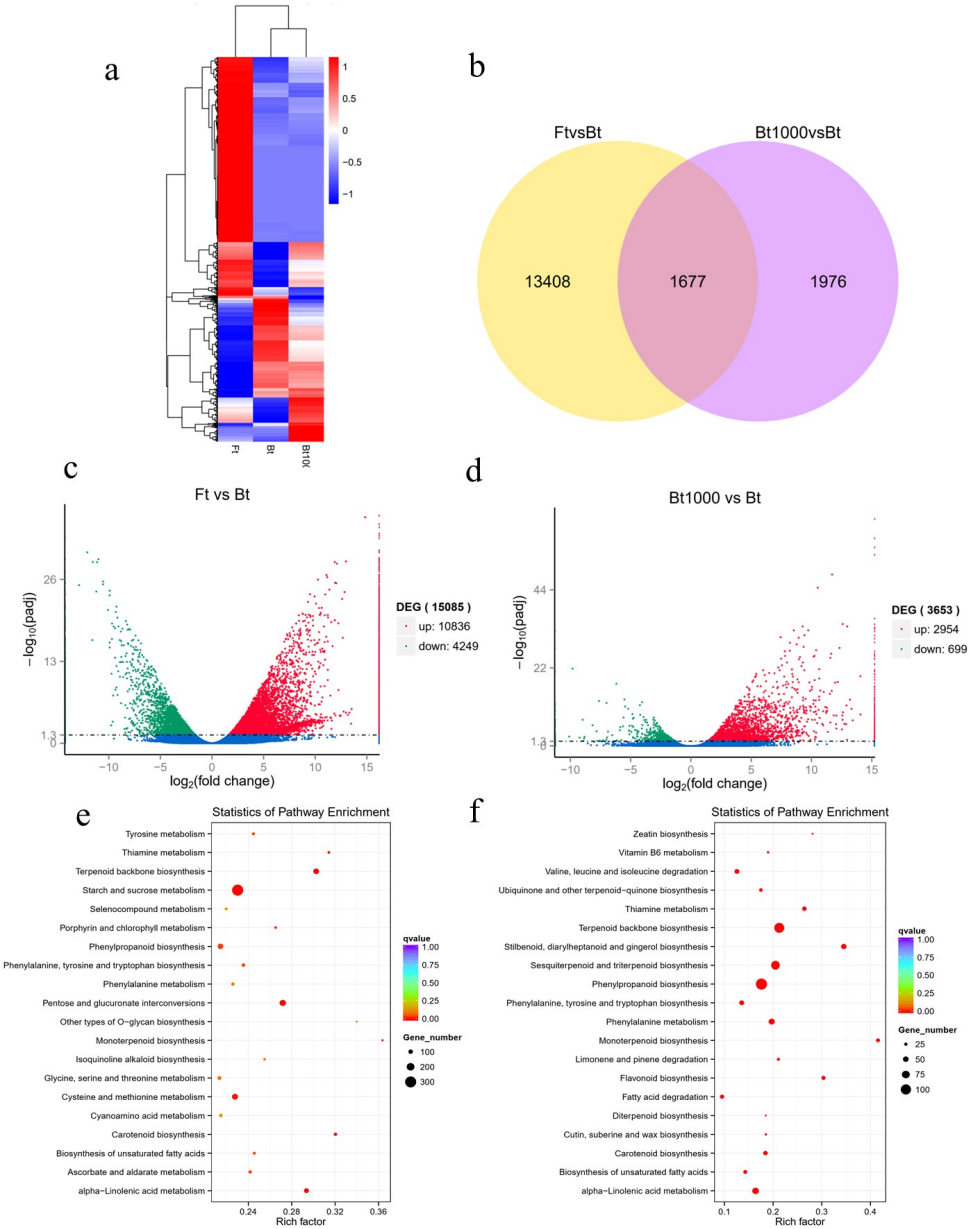
Transcription profiles of *Lonicera macranthoides* Hand.-Mazz. (a): The cluster heat map for differentially expressed genes (DEGs). (b): Venn diagram showing the distribution of DEGs from Bt, Ft and Bt1000. (c): Volcano plot of differentially expressed genes between Ft and Bt. (d): Volcano plot of differentially expressed genes between Bt1000 and Bt. (e): KEGG enrichment analysis of the differentially accumulated metabolites (DAMs) between Ft and Bt. (e): Kyoto Encyclopedia of Genes and Genomes Ortholog database (KEGG) enrichment analysis of the DAMs between Bt1000 and Bt. Ft indicates the flowering type, Bt indicates closed bud type and Bt1000 indicates MeJA-treated closed bud type.

The FPKM method [35] was used to calculate the expression of all unigenes to remove the effects of length differences and sequencing depth. DEGs are defined as genes that are significantly enriched or depleted in one sample relative to another by DESeq [38] (qvalue < 0.005 and log2 (fold change) > 1). The results of transcriptome analysis showed that the gene expression levels were different in three lines of samples of *L*. *macranthoides*. The clustering heat map shows that there were obvious differences in gene expression in them. There was overlap between Bt1000 and Bt with lower expression levels, while there was almost no overlap between Bt and Ft (Fig 6a). The Venn diagram of the differential genes shows the number of common and specific genes of three lines of samples compared in pairs. From Fig 6b, the expression levels of 1, 677 genes were different in 3 different types of samples, with 13, 408 and 1, 976 having specific differences, respectively. The volcano map (Fig 6c) revealed that the expression levels of 10, 836 and 4, 249 DEGs were higher or lower in Ft than in Bt, while the expression levels of 2, 954 and 699 DEGs were higher or lower in Bt1000 than in Bt. The KEGG enrichment analysis of the DAMs indicated that most of the identified DEGs act on multiple metabolic processes related to terpenoid backbone biosynthesis, phenylpropanoid biosynthesis, sesquiterpenoid and triterpenoid biosynthesis and stilbenoid, diarylheptanoid and gingerol biosynthesis in Bt1000 vs Bt (Fig 6e). The DEGs of Ft vs Bt were enriched on terpenoid backbone biosynthesis, pentose and glucuronate interconversions and starch and sucrose metabolism (Fig 6f). The phenylpropanoid biosynthesis which we focused on enriched 73 DEGs. In total, 6 DEGs were selected for qRT-PCR quantification experiments to verify the accuracy of transcriptome sequencing. The results indicated that the expression patterns from qRT-PCR testing were well correlated with sequencing results (S1 Fig), indicating the reliability of the transcriptomic data.

### Differentially expressed genes related to flowering in three lines of samples (Bt, Ft, Bt1000) of *L*. *macranthoides*

Ft is prone to corolla dehiscence in the florescence period, and this period is relatively short, generally 3–7 days. If the florescence period is overcast and rainy, it is easy to fall flowers, which is inconvenient for harvesting and processing, and affects the yield and quality. The new cultivar ‘Bt’ does not crack in its corolla at the flowering stage, and its flower buds are all in the shape of rods. The flower buds are orderly, and the flowering period lasts for 15–20 days. Moreover, the yield and CGAs content are obviously higher than Ft. In order to investigate whether flower corolla dehiscence is the direct factor affecting the CGAs biosynthesis in *L*. *macranthoides*, we started with the regulation of flowering related genes and conducted a combined analysis with the metabolome method. To explore the flowering molecular mechanism of *L*. *macranthoides*, we further investigated the changes in gene expression profiles among the three lines of samples. Under MeJA treatment, 16 DEGs were labeled flowering-related genes (*FRGs*) between Bt1000 and Bt and included members of the embryonic flower (*EMF*), cycling dof factors (*CDF*), and early flowering (*ELF*) families, as well as MADS-box transcription factor (*MADS-box*). Most of them were upregulated under MeJA treatment (S2 Table), indicating that these *FRGs* mainly respond to MeJA treatment through positive feedback regulation. In our experiment, the transcriptional abundance of flowering-related genes was significantly altered under MeJA treatment. Some genes in the CDF family, especially i1_LQ_LMFt_c43539/f1p0/1681 and i1_LQ_LMFt_c60085/f1p0/1664, which were upregulated by 125-fold and 106-fold, respectively, were heavily programmed and highly induced under MeJA treatment (S2 Table). However, the expression abundance of *MADS-box*-related genes (i2_LQ_LMFt_c19027/f1p2/2455 and i1_LQ_LMFt_c60228/f1p9/1090) decreased significantly, indicating that this type of gene had a negative feedback regulation on the flowering of L. *macranthoides*. A total of 38 DEGs, including the *MADS-Box*, *FRI*, *FCA*, *ELF* and *SVP* families, were screened from Ft and Bt of *L*. *Macranthoides*. Among these DEGs, 29 DEGs were upregulated in Ft, and 13 DEGs were not expressed in Bt. I1_LQ_LMFt_c51599/f1p8/1110 (*MADS-box*) and i3_LQ_LMFt_c13420/f1p0/3016 (*ELF*) were upregulated by 886-fold and 654-fold (S3 Table), respectively. The above results indicate that *MADS-Box* and other genes play an important role in regulating the flowering of *L*. *macranthoides*, and further research will be carried out.

### DEG and DAM in the chlorogenic acids biosynthesis pathway between Bt and Ft

**Fig 7:**
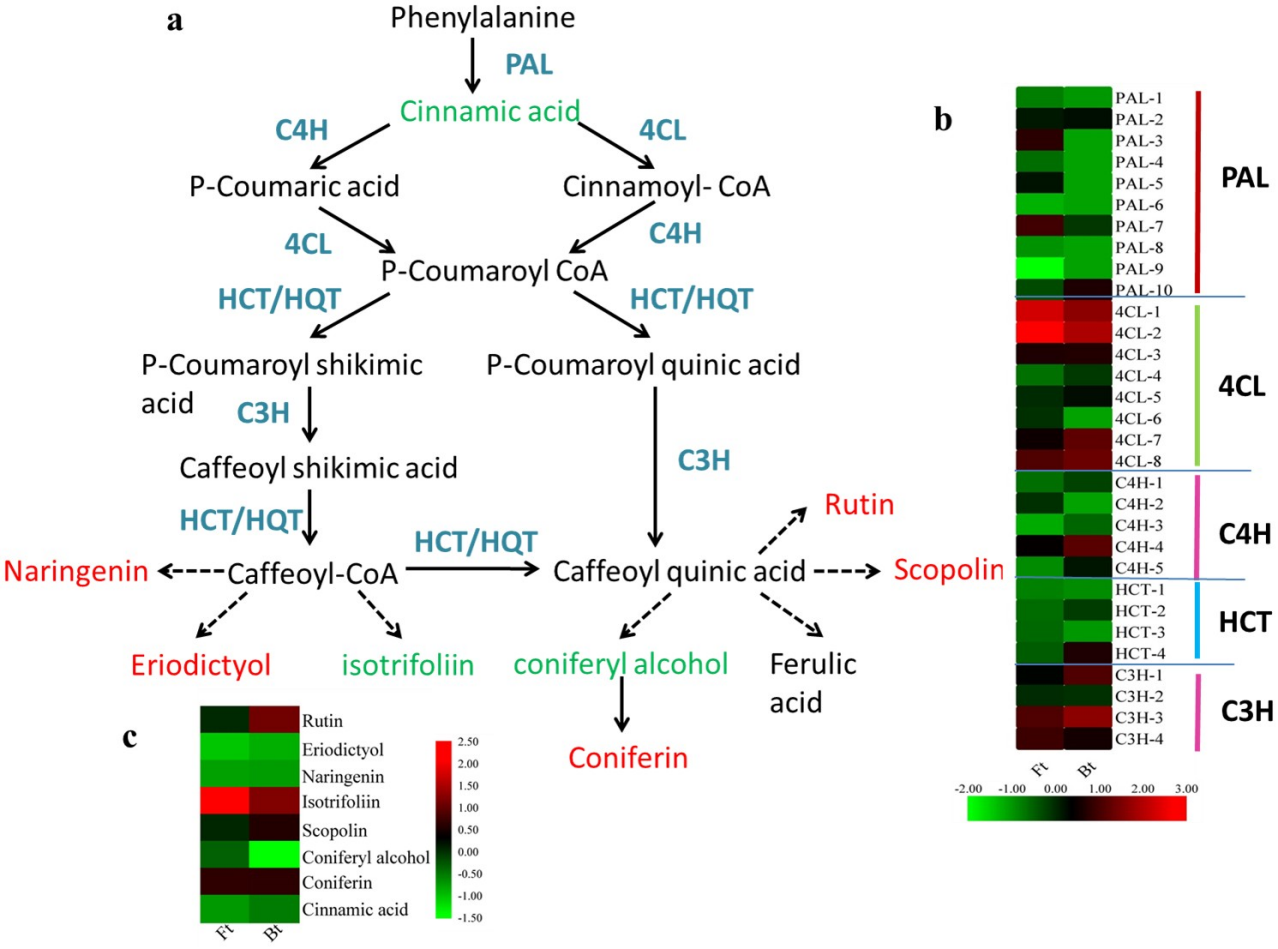
Biosynthetic pathway of chlorogenic acids. (a): Metabolic pathway map of integrative chlorogenic acids biosynthesis in *L*. *macranthoides*. Red indicates an increase in content, and the blue indicates a decrease in content. (b): Heat map of changes in transcripts in chlorogenic acids biosynthesis. (c): Heat map of changes in metabolites in chlorogenic acids biosynthesis. Upregulated (red) and downregulated (green) genes and metabolites are indicated. Ft indicates the flowering type, Bt indicates closed bud type and Bt1000 indicates MeJA-treated closed bud type.

It has been reported that CGAs is the most interesting of the biologically active ingredients of *L*. *macranthoides* [1]. As the content of CGAs in Ft and Bt was significantly different over the course of this analysis, KEGG pathways involved in CGAs biosynthesis were further analyzed in this study. CGAs biosynthesis mainly involves the phenylalanine biosynthesis pathway, which is very complex and involves the regulation of various enzymes, such as *PAL*, *4CL*, *C4H*, *HCT* and *C3H* (Fig 7a). According to KEGG enrichment results and FPKM values, 30 key enzyme genes differentially expressed in CGAs biosynthesis were successfully screened from DAMs, including *PAL* (10), *4CL* (8), *C4H* (5), *HCT* (4), and *C3H* (3) (S4 Table). The data indicated that all CGAs biosynthesis pathway genes showed significantly higher levels of gene transcripts in flowering type (Ft). Six of the ten *PAL* genes involved in the synthesis of cinnamic acid were not expressed in closed bud type (Bt), two of which exhibited a higher level of expression in Ft. In *4CL* genes, i1_LQ_LMFt_c23140/f1p7/1891 was not expressed in Bt. The expression level of most of the *4CL* genes in Ft was upregulated, with the highest difference being a 24-fold increase. The *C4H-2* was not expressed in Bt, while the other *C4H* were upregulated in Ft. Especially in *HCT* genes, there were two genes with high differential expression multiples, up to 288 and 775 times (Fig 7b). However, interestingly, although these genes were upregulated in Ft, the ion abundance of most CGAs in Ft were significantly lower than that of Bt (Table 3). We know that in the phenylalanine biosynthesis pathway, the synthesis reaction does not stay on CGAs, and the next step product is synthesized under the catalysis of other enzymes. In our experiment, a total of 8 DAMs were confirmed based on UPLC-MS, as shown in Fig 7c. Apart from cinnamic acid, the 7 DAMs are downstream of the phenylalanine biosynthesis pathway. Upstream of CGA synthesis, the ion abundance of eriodictyol and naringenin in Ft was significantly higher than that in Bt. Compared with Bt, the ion abundance of Rutin, Coniferin and Scopolin in Ft was up-regulation (Fig 7c). To some extent, although the upstream genes of CGAs biosynthesis are upregulated in Ft, due to the generation of caffeoyl-COA and CGAs are catalyzed by corresponding enzymes to synthesize downstream substances, resulting in the ion abundance of CGAs in Ft being significantly lower than that of Bt.

**Table 3:** Ion abundance of chlorogenic acids in Bt, Ft and Bt1000.

Metabolite	Ion abundance			Fold change	
	Bt	Bt1000	Ft	Ft vs Bt	Bt1000 vs Bt
Chlorogenic acid	3.60E+07	2.94E+07	3.10E+07	0.86	0.82
Chlorogenic acid methyl ester	1.50E+06	1.90E+06	1.00E+06	0.67	1.27
Cryptochlorogenic acid	1.95E+07	2.18E+07	7.36E+06	0.38	1.11
Neochlorogenic acid	9.95E+06	5.99E+06	8.07E+06	0.81	0.60
Isochlorogenic acid A	8.48E+06	9.38E+06	1.09E+07	1.29	1.11
Isochlorogenic acid C	8.36E+06	9.76E+06	1.05E+07	1.26	1.17
Total	8.39E+07	7.82E+07	6.89E+07	0.82	0.93

Note: Ft indicates the flowering type, Bt indicates closed bud type and Bt1000 indicates MeJA-treated closed bud type.

### DEG and DAM in the chlorogenic acids biosynthesis pathway between Bt and Bt1000

In the preliminary study of our group, it was found that MeJA treatment at a certain concentration could effectively promote the corolla opening of Bt. To determine whether MeJA affects the synthesis of CGAs and the relationship between flowering and synthesis of CGAs, we treated the buds of Bt with MeJA and carried out studies by means of metabolomics and transcriptomics analysis methods. In our study, the genes related to CGAs biosynthesis in Bt showed an upregulation under the treatment with MeJA. Among these DEGs, i2_LQ_LMFt_c5476/f1p0/2700 (annotated as *PAL*) and i1_LQ_LMFt_c48921/f1p17/1795 (annotated as *C3H*) were upregulated by 17-fold and 37-fold (S5 Table), respectively. We detected that the upstream substance in Bt1000 was higher than that in Bt. However, the ion abundance of CGAs was slightly lower than that of Bt. We found that the alterations of Bt1000 compared to Bt were increased in cinnamic acid and coniferin, and decreased in caffeic acid and coniferyl alcohol (S6 Table). From the above results, MeJA leads to the activation of downstream genes in the phenylalanine biosynthesis pathway, and more substances flow downstream. However, whether MeJA and tube cracking have direct effects on the biosynthesis of CGAs needs to be further studied.

### Correlation analysis between DEGs and DAMs in other metabolic pathways

According to the results of differential metabolite analysis in this study and combined with the conjoint analysis of DEGs and DAMs results, the genes and metabolites in the same group were simultaneously mapped to the KEGG pathway map to better understand the relationship between genes and metabolites. We found that the alterations of Ft compared to Bt were also associated with 12 metabolic pathways, including pentose and glucuronate interconversions, ascorbate and aldarate metabolism, ubiquinone and other terpenoid-quinone biosynthesis, phenylpropanoid biosynthesis and so on (S7 Table). The alterations of Bt compared to Bt1000 were also associated with 20 metabolic pathways (S8 Table). In the correlation analysis, D-galacturonic acid, cinnamic acid and succinic acid were highly correlated with the corresponding metabolic pathways, which also provides a new perspective for our subsequent research.

### Discussion

In general, *L*. *macranthoides*’s buds or open flowers are used as medicine [2]. The flowering type (Ft)’s bud period was shorter, and after corolla dehiscence, the flowers were easy to drop in cloudy and rainy days which brings considerable trouble to their harvest [42]. The closed bud type (Bt) is a new cultivar with non—cracking corolla, and the CGAs abundance is significantly higher than that in Ft; therefore, Bt is recommended in production. With the flowering process, CGAs content decreases significantly. The abundance of active components and volatile compounds are closely correlated with floral developmental stages [43]. Moreover, MeJA can effectively regulate corolla opening. The quality of herbal medicine has been very difficult to control and evaluate primarily because of the complexity and incomplete knowledge of the active medicinal compounds [42]. To explore the synthesis mechanism of CGAs in different flowering types and MeJA-treated Bt, a comprehensive analysis of the transcriptome and metabolome was performed.

CGAs is the most interesting of the biologically active ingredients of *L*. *macranthoides* [10]. CGAs are the primary phenylpropanoid generated from the shikimic acid pathways with high antioxidant activities. In our results, 341 metabolites were detected and quantified by UHPLC-ESI-MS/MS with a self-built database. We profiled the chemical compositions of *L*. *macranthoides* in a systematic and comprehensive way, which provided a reference for their utilization in the future. Studies on *L*. *japonica*, coffee, artichoke and other plants have revealed that PAL, C4H and 4CL are the common enzymes in the upstream CGAs metabolic pathway [2, 41, 44, 45]. DEGs related to CGAs biosynthesis were identified between Bt and Ft, including ten *PAL*, eight *4CL*, five *C4H*, four *C3H* and four *HCT* genes. Among them, we revealed that most of the enzyme genes in the CGAs biosynthetic pathway were highly active in Ft. Similarly, in terms of metabolites, the upstream substances of CGAs, coumaric acid and cinnamic acid, were upregulated in Ft. In contrast, the content of CGAs in the phenylalanine synthesis pathway was significantly different. The CGAs in Bt was significantly higher than that in Ft. On the basis of previous research, we drew a putative road map of the phenylalanine biosynthesis pathway. Transcriptomics integrated with metabolomics revealed that most components of downstream material showed drastically higher abundances in Ft than in Bt, accompanied by significant reductions in the CGAs. The above results show that in Ft, the particle flow does not stay at the CGAs stage but is synthesized downstream at a high rate. Similar results have also been reported in ginkgo and peanut species [46, 47]. An organism is complex. A metabolite may be regulated by multiple genes, and a gene may regulate multiple metabolites, so there may not be one-to-one relationship between metabolites and genes.

The correlation between flowering and chlorogenic acids biosynthesis is also discussed. MeJA is an endogenous growth regulator in plants and has a wide range of physiological functions. From previous research results, MeJA can regulate the synthesis of flavonoids in plants [44, 47]. However, the effect of MeJA on the biosynthesis of phenolic acids is still unclear. At the same time, whether there is any correlation between flowering and chlorogenic acids biosynthesis has not been reported. Most of the 16 *FRGs* were upregulated under MeJA treatment, indicating that these *FRGs* mainly respond to MeJA treatment through positive feedback regulation. A total of 38 DEGs, including the *MADS-Box*, *FRI*, *FCA*, *ELF* and SVP families, were found in the typical flower type (Ft) and the close bud type (Bt) of *L*. *macranthoides*. Among these DEGs, 28 DEGs were upregulated in Ft, and 13 DEGs were not expressed in Bt. *MADS-box* family genes in plants are important transcriptional regulators and are involved in the regulation of flower organ development and flowering time [48–50]. Different *MADS-Box* family genes showed different expression trends in Ft and Bt, indicating that this gene family had both positive and negative regulation on the flowering of *L*. *macranthoides*. From the results of this study, we screened 14 genes related to ‘early flowering’, one of which was mostly upregulated in Ft. In particular, the expression of this kind of gene also showed an upregulation in MeJA-treated samples, indicating that the study of this kind of gene will provide us with a new idea for the analysis of *L*. *macranthoides* flowering time. The above results indicated that the expression of genes regulating flowering was significantly different in different samples. However, by analyzing the metabolites of the three lines of samples, no direct effect of flowering genes on chlorogenic acids biosynthesis was found. We usually think that flowering affects the content of CGAs in *L*. *macranthoides*, but no direct evidence was found to prove this. Studies have shown that the endogenous hormone ethylene plays a key role in the flowering and aging process of plants [51]. In subsequent studies, the correlation between hormone metabolism, flowering and biosynthesis of active substances will be studied by combining transcriptomics and metabolites.

Currently, studies have shown that genes mentioned in this article are affected by MeJA-treated, and the abundance of metabolites also varies, but the mechanism of action is unclear. Therefore, we plan to focus on the molecular mechanism of MeJA regulating flowering and CGAs biosynthesis. At the same time, the molecular regulation mechanism of CGAs biosynthesis will be further explored to provide a better theoretical basis for the breeding of *L*. *macranthoides* varieties and the preservation of excellent genes.

### Conclusion

Through conjoint metablomic and transcriptomic analysis of Ft, Bt and Bt1000, we concluded that: (1) 17, 061 DEGs and 341 DAMs among Bt, Ft and Bt1000 were identified. (2) Based on correlation analyses between DEGs and DAMs, we found 30 candidate genes for the accumulation of CGAs. We further identified approximately 54 candidate genes for regulating the flowering. (3) The redirection of metabolic flux may contribute to increased accumulation of CGAs in Bt compared to Ft. MeJA treatment may leads to the activation of downstream genes in the phenylalanine biosynthesis pathway. However, whether the MeJA and flower tube cracking have direct effects on the accumulation of CGAs needs to further studied. These results provide novel insights into CGAs biosynthesis. The candidate genes for CGAs biosynthesis and flowering presented here represent a valuable data set for future functional studies.

## Supporting information

S1 Table Primer sequence for qRT-PCR. (XLSX)

S2 Table Flowering related DEGs between Bt1000 and Bt. (XLSX)

S3 Table Flowering related DEGs between Ft and Bt. (XLSX)

S4 Table Different expressed genes related to chlorogenic acids biosynthesis between Ft and Bt. (XLSX)

S5 Table Different expressed genes related to chlorogenic acids biosynthesis between Bt1000 and Bt. (XLSX)

S6 Table Ion abundance of chlorogenic acids biosynthesis downstream substances between Bt1000 and Bt. (XLSX)

S7 Table DEGs and DAMs involved in other metabolic pathways between Bt and Ft. (XLSX)

S8 Table DEGs and DAMs involved in other metabolic pathways between Bt1000 and Bt. (XLSX)

S9 Table DAMs of Bt vs Ft. (XLSX)

S10 Table DAMs of Bt vs Bt1000. (XLSX)

S1 Fig Validation of the transcription levels for selected DEGs by qRT-PCR. (XLSX)

## Abbreviations

*4CL*: 4-coumarate coenzyme A ligase
Bt: closed bud type
Bt1000: MeJA-treated
*C3H*: coumarate 3-hydroxylase
*C4H*: cinnamate 4-Hydroxylase
CGAs: chlorogenic acids
DAM: differentially accumulated metabolite
DEG: differentially expressed genes
*ELF*: early flowering
*EMF*: embryonic flower
*FRGs*: flowering-related genes
Ft: flowering type
*HCT*: hydroxycinnamoyl transferase
*HQT*: hydroxycinnamoyl CoA quinate hydrocycinnamoyl transferase
*MADS-box*: MADS-box transcription factor
MeJA: jasmonic acid methyl ester
OPLS-DA: Orthogonal partial least squares discriminant analysis
*PAL*: phenylalanine ammonia lyase
PCA: principal components analysis

## References

[1] Xiao C, Wang Z, Tian L. Several proposed amendments to the criteria for Lonicera japonica and Lonicera macranthoides in "Chinese Pharmacopoeia". China Journal of Chinese Materia Medica. 2011; 3:1406–1407.

[2] Chen Z, Tang N, You Y, Lan J, Liu Y, Li Z. Transcriptome Analysis Reveals the Mechanism Underlying the Production of a High Quantity of Chlorogenic Acid in Young Leaves of Lonicera macranthoides Hand.-Mazz. PLoS ONE. 2015; 10(9): e0137212. 10.1371/journal.pone.0137212 26381882 PMC4575056

[3] LiKun Z, Zhen Z, Xia-Ming J, Yishan Z, Xi C, Zheng F, et al. Absorbed plant MIR2911 in honeysuckle decoction inhibits SARS-CoV-2 replication and accelerates the negative conversion of infected patients. Cell Discovery. 2020; 6:54. 10.1038/s41421-020-00197-3 32802404 PMC7406496

[4] Rai A, Kamochi H, Suzuki H, Nakamura M, Takahashi H, Hatada T, et al. De novo transcriptome assembly and characterization of nine tissues of Lonicera japonica to identify potential candidate genes involved in chlorogenic acid, luteolosides, and secoiridoid biosynthesis pathways. Journal of Natural Medicines. 2017; 71(1):1–15. 10.1007/s11418-016-1041-x 27629269 PMC5214891

[5] Park K, Park H, Nagappan A, Hong GE, Yumnam S, Lee HJ, et al. Polyphenolic compounds from Korean Lonicera japonica Thunb. Induces apoptosis via AKT and caspase cascade activation in A549 cells. Oncol Lett. 2017;13(4):2521–30. 10.3892/ol.2017.5771 28454429 PMC5403260

[6] Jianjun L, Chenglin Y, Cuifang C. Comparative transcriptomics analysis revealing flower trichome development during flower development in two Lonicera japonica Thunb. cultivars using RNA-seq. BMC plant Biology. 2020, 20: 341. 10.1186/s12870-020-02546-6 32680457 PMC7368687

[7] Kishimoto N KY, Iwai K, Mochida K, Fujita T. In vitro antibacterial, antimutagenic and anti-influ-enza virus activity of caffeic acid phenethyl esters. Biocontrol science. 2005; 10: 155–161.

[8] Zhang B, Nan TG, Xin J, Zhan ZL, Kang LP, Yuan Y, et al. Development of a colloidal gold-based lateral flow dipstick immunoassay for rapid detection of chlorogenic acid and luteoloside in Flos Lonicerae Japonicae. Journal of Pharmaceutical and Biomedical Analysis. 2019; 170: 83–8. 10.1016/j.jpba.2019.03.035 30909057

[9] Park C, Lee WS, Han MH, Song KS, Hong SH, Nagappan A, et al. Lonicera japonica Thunb. Induces caspase-dependent apoptosis through death receptors and suppression of AKT in U937 human leukemic cells. Phytother Research. 2018; 32 (3):504–13. 10.1002/ptr.5996 29193390

[10] Han JM, Kim MH, Choi YY, Lee H, Hong J, Yang WM. Effects of Lonicera japonica Thunb. On type 2 diabetes via PPAR-gamma activation in rats. Phytother Research. 2015; 29 (10):1616–21. 10.1002/ptr.5413 26174209

[11] Zhang T, Liu H, Bai X, Liu P, Yang Y, Huang J, et al. Fractionation and antioxidant activities of the water-soluble polysaccharides from Lonicera japonica Thunb. International journal of biological macromolecules. 2020; 151:1058–66. 10.1016/j.ijbiomac.2019.10.147 31739015

[12] Clifford MN. Diet-derived phenols in plasma and tissues and their implications for health. Planta Medica. 2004; 70: 1103–1114. 10.1055/s-2004-835835 15643541

[13] Chen QZ, Lin RC, Wang GL, Li FM. Studies on chemical constituents of the extract of Lonicera japonica. Zhong Yao Cai. 2010; 33: 920–922. 21049617

[14] Zhang C, Yin Z, Ye W, Guan Y, Guo L, Zhang J, et al. Chemical constituents from stems of Lonicera japonica. Zhongguo Zhong Yao Za Zhi. 2009; 34: 3051–3053. 20222422

[15] Kong D, Li Y, Bai M, Deng Y, Liang G, Wu H. A comparative study of the dynamic accumulation of polyphenol components and the changes in their antioxidant activities in diploid and tetraploid Lonicera japonica. Plant Physiology and Biochemistry. 2017; 112:87–96. 10.1016/j.plaphy.2016.12.027 28049060

[16] Li Y, Kong D, Wu H. Comprehensive chemical analysis of the flower buds of five Lonicera species by ATR-FTIR, HPLC-DAD, and chemometric methods. Revista Brasileira de Farmacognosia. 2018; 28 (5):533–541.

[17] Clifford MN. Chlorogenic acids and other cinnamates–nature, occurrence, dietary burden, absorption and metabolism. Journal of the Science of Food and Agriculture. 2020; 80: 1033–1043.

[18] Nardini M, Cirillo E, Natella F, Scaccini C. Absorption of phenolic acids in humans after coffee consumption. Journal of Agricultural and Food Chemistry. 2002; 50: 5735–5741. 10.1021/jf0257547 12236707

[19] Paul A H, Vincent J H, Paiva Nancy L. Overexpression of L-Phenylalanine Ammonia-Lyase in Transgenic Tobacco Plants Reveals Control Points for Flux into Phenylpropanoid Biosynthesis. Plant Physiology. 1996; 112: 1617–1624. 10.1104/pp.112.4.1617 12226468 PMC158095

[20] Falcone Ferreyra ML, Rius S, Casati P. Flavonoids: Biosynthesis, biological functions, and biotechnological applications. Front of Plant Science. 2012; 3: 222. 10.3389/fpls.2012.00222 PMC3460232 23060891

[21] Chen J, Tang XH, Ren CX, Wei B, Wu YY, Wu QH, et al. Full-length transcriptome sequences and the identification of putative genes for flavonoid biosynthesis in safflower. BMC Genomics. 2018;19 (1):548–60. 10.1186/s12864-018-4946-9 30041604 PMC6057038

[22] Abdulrazzak N, Pollet B, Ehlting J, Larsen K, Asnaghi C, Ronseau S, et al. A coumaroyl-ester-3-hydroxylase insertion mutant reveals the existence of nonredundant meta-hydroxylation pathways and essential roles for phenolic precursors in cell expansion and plant growth. Plant Physiology. 2006; 140:30–48. 10.1104/pp.105.069690 16377748 PMC1326029

[23] Schoch G, Goepfert S, Morant M, Hehn A, Meyer D, Ullmann P, et al. CYP98A3 from Arabidopsis thaliana is a 30-hydroxylase of phenolic esters, a missing link in the phenylpropanoid pathway. The Journal of Biological Chemistry. 2001; 276: 36566–36574. 10.1074/jbc.M104047200 11429408

[24] Comino C, Hehn A, Moglia A, Menin B, Bourgaud F, Lanteri S, et al. The isolation and mapping of a novel hydroxycinnamoyltransferase in the globe artichoke chlorogenic acid pathway. BMC Plant Biology. 2009; 9:30. 10.1186/1471-2229-9-30 19292932 PMC2664813

[25] Comino C, Lanteri S, Portis E, Acquadro A, Romani A, Hehn A, et al. Isolation and functional characterization of a cDNA coding a hydroxycinnamoyltransferase involved in phenylpropanoid biosynthesis in Cynaracardunculus L. BMC Plant Biology. 2007; 7:14. 10.1186/1471-2229-7-14 17374149 PMC1847684

[26] Hoffmann L, Maury S, Martz F, Geoffroy P, Legrand M. Purification, cloning, and properties of an acyltransferase controlling shikimate and quinate ester intermediates in phenylpropanoid metabolism. The Journal of Biological Chemistry. 2003; 278: 95–103. 10.1074/jbc.M209362200 12381722

[27] Strack D, Gross W. Properties and Activity Changes of Chlorogenic Acid:Glucaric Acid Caffeoyl-transferase From Tomato (Lycopersicon esculentum). Plant Physiology. 1990; 92: 41–47. 10.1104/pp.92.1.41 16667263 PMC1062245

[28] Zhen Z, Changping T, Ya Zh, Chenzhiyu L, Xi L, Qiang Y, et al. BMC Plant Biology, 2020, 20:129. 10.1186/s12870-020-02344-0 32220242 PMC7099803

[29] Jie G, Rui R, Yonglu W, Jianpeng J, Sagheer A, Chuqiao L, et al. Comparative Metabolomic Analysis Reveals Distinct Flavonoid Biosynthesis Regulation for Leaf Color Development of Cymbidium sinense ‘Red Sun’. International Journal of Molecular Sciences. 2020, 21, 1869. 10.3390/ijms21051869 PMC7084835 32182912

[30] Jiang C, Jie W, Rui W, Bin X, Chaoxiang R, Qianqian L, et al. Integrated metabolomics and transcriptome analysis on flavonoid biosynthesis in safflower (Carthamus tinctorius L.) under MeJA treatment. BMC Plant Biology, 2020, 20:353 10.1186/s12870-020-02554-6 32727365 PMC7391820

[31] Yuan H, Zeng X, Shi J, Xu Q, Wang Y, Jabu D, et al. Time-course comparative metabolite profiling under osmotic stress in tolerant and sensitive Tibetian hulless barley. BioMed Research International. 2018; 9415409: 1–12. 10.1155/2018/9415409 PMC6323448 30671479

[32] Wang Z, Cui Y, Vainstein A, Chen S, Ma H. Regulation of fig (Ficus carica L.) fruit color: metabolomic and transcriptomic analyses of the flavonoid biosynthetic pathway. Front Plant Science. 2017; 8:1990. 10.3389/fpls.2017.01990 29209349 PMC5701927

[33] Wang Z, Cui Y, Vainstein A, Chen S, Ma H. Regulation of fig (Ficuscarica L.) fruit color: Metabolomic and Transcriptomic analyses of the flavonoid biosynthetic pathway. Front Plant Science. 2017; 8: 1990–2004. 10.3389/fpls.2017.01990 PMC5701927 29209349

[34] Grabherr M G, Haas B J, Yassour M, et al. Full-length transcriptome assembly from RNA-Seq data without a reference genome. Nature Biotechnology. 2011; 29, 644–652. 10.1038/nbt.1883 21572440 PMC3571712

[35] Trapnell C, Williams B A, Pertea G. Transcript assembly and quantification by RNA-Seq reveals unannotated transcripts and isoform switching during cell differentiation. Nat Biotech. 2010; 28, 511–515. 10.1038/nbt.1621 20436464 PMC3146043

[36] Kanehisa M, Araki M, Goto S. KEGG for linking genomes to life and the environment. Nucleic Acids research. 2008; 36: D480–D484. 10.1093/nar/gkm882 18077471 PMC2238879

[37] Conesa A, Götz S, García-Gómez JM, Terol J, Talón M, Robles M. Blast GO: a universal tool for annotation, visualization and analysis in functional genomics research. Bioinformatics. 2005; 21(18):3674–6. 10.1093/bioinformatics/bti610 16081474

[38] Anders S, Huber W. Differential expression analysis for sequence count data. Genome Biology. 2010;. 10.1186/gb-2010-11-10-r106 20979621 PMC3218662

[39] Young MD, Wakefield MJ, Smyth GK. Gene ontology analysis for RNA-seq: accounting for selection bias. Genome Biology. 2010; 10.1186/gb-2010-11-2-r14 20132535 PMC2872874

[40] Mao X, Cai T, Olyarchuk J G. Automated genome annotation and pathway identification using the KEGG Orthology (KO) as a controlled vocabulary. Bioinformatics. 2005; 21: 3787–3793. 10.1093/bioinformatics/bti430 15817693

[41] He L, Xu X, Li Y, Li C, Zhu Y, Yan H, et al. Transcriptome analysis of buds and leaves using 454 pyrosequencing to discover genes associated with the biosynthesis of active ingredients in Lonicera japonica Thunb. PLoS One. 2014 8: e62922. 10.1371/journal.pone.0062922 PMC3636143 23638167

[42] Yuan Y, Lipu S, Minhui L, Guiming L, Yanan C, Luyu M, et al. Genetic variation and metabolic pathway intricacy govern the active compound content and quality of the Chinese medicinal plant Lonicera japonica thumb. BMC Genomics. 2012; 13:195. 10.1186/1471-2164-13-195 22607188 PMC3443457

[43] Geng S, Ning X, Wu H, Lin H, Zhao S, Xu H. The Structure of Flower in Different Developmental Stages in Relation to the Varieties of Chlorogenic Acid Content in Lonicera confusa. Acta Botanica Yunnanica. 2005; 27(3): 279–287

[44] Sonnante G, DAmore R, Blanco E, Pierri CL, De Palma M, Luo J, et al. Novel hydroxycinna-moyl-coenzyme A quinate transferase genes from artichoke are involved in the synthesis of chlorogenic acid. Plant Physiology. 2010; 153: 1224–1238. 10.1104/pp.109.150144 20431089 PMC2899911

[45] Comino C, Hehn A, Moglia A, Menin B, Bourgaud F, Lanteri S, et al. The isolation and mapping of a novel hydroxycinnamoyltransferase in the globe artichoke chlorogenic acid pathway. BMC Plant Biology. 2009; 9: 30. 10.1186/1471-2229-9-30 19292932 PMC2664813

[46] Liyun W, Yong L, Liying Y, Yue L, Pandey Manish K, et al. Transcriptome and metabolome reveal redirection of flavonoids in a white testa peanut mutant. BMC Plant Biology. 2020; 20:161. 10.1186/s12870-020-02383-7 32293272 PMC7161308

[47] Jie M, Bo W, Guo H, Yu W, Xianfeng T, Shumin W, et al. Metabolomics Integrated with Transcriptomics Reveals Redirection of the Phenylpropanoids Metabolic Flux in Ginkgo biloba. Journal of Agricultural and Food Chemistry. 2019; 67, 3284–3291 10.1021/acs.jafc.8b06355 30802049

[48] Jiang C, Jie W, Rui W, Bin X, Chaoxiang R, Qianqian L, et al. Integrated metabolomics and transcriptome analysis on flavonoid biosynthesis in safflower (Carthamus tinctorius L.) under MeJA treatment. BMC Plant Biology. 2020; 20:353 10.1186/s12870-020-02554-6 32727365 PMC7391820

[49] Colombo M, Masiero S, Vanzulli S. AGL23, a type I MADS-box gene that controls female gametophyte and embryo development in Arabidopsis. Plant Journal. 2008, 54(6): 1037–1048. 10.1111/j.1365-313X.2008.03485.x 18346189

[50] Gunter T, Annette B, Alexandra D R. A short historyof MADS-box genes in plants. Plant Molecular Biology. 2000; 42(1): 115–149. 10688133

[51] Woltering EJ, Doorn WGV. Role of ethylene in senescence of petals-morphological and taxonomical relationships. Journal of Experimental Botany. 1988; 39 (11):1605–1616.

